# Population Dynamics P System (PDP) Models: A Standardized Protocol for Describing and Applying Novel Bio-Inspired Computing Tools

**DOI:** 10.1371/journal.pone.0060698

**Published:** 2013-04-09

**Authors:** Maria Àngels Colomer, Antoni Margalida, Mario J. Pérez-Jiménez

**Affiliations:** 1 Department of Mathematics, University of Lleida, Lleida, Spain; 2 Division of Conservation Biology, Institute of Ecology and Evolution, University of Bern, Bern, Switzerland; 3 Research Group on Natural Computing, Department of Computer Science and Artificial Intelligence, University of Sevilla, Sevilla, Spain; Universitat Rovira i Virgili, Spain

## Abstract

Today, the volume of data and knowledge of processes necessitates more complex models that integrate all available information. This handicap has been solved thanks to the technological advances in both software and hardware. Computational tools available today have allowed developing a new family of models, known as computational models. The description of these models is difficult as they can not be expressed analytically, and it is therefore necessary to create protocols that serve as guidelines for future users. The Population Dynamics P systems models (PDP) are a novel and effective computational tool to model complex problems, are characterized by the ability to work in parallel (simultaneously interrelating different processes), are modular and have a high computational efficiency. However, the difficulty of describing these models therefore requires a protocol to unify the presentation and the steps to follow. We use two case studies to demonstrate the use and implementation of these computational models for population dynamics and ecological process studies, discussing briefly their potential applicability to simulate complex ecosystem dynamics.

## Introduction

Changes in the dynamics of ecological communities depend heavily on interactions between populations of predators and their prey, and the pressure that they place on ecosystems [1]–[3]. Quantifying the interactions between species is essential to understanding how ecological communities are organized and how they can respond to human intervention [4]–[5]. In recent years, a significant amount of information has been validated and contrasted allowing the analysis of the interactions, as well as of interspecific and intraspecific relationships of ecosystems, highlighting the complexity of the problems [6]–[9]. The next step to improving the understanding of the complexity of the network structure in ecosystems is to define a computational model to perform virtual experiments using simulators that resemble as closely as possible the behaviour and functioning of the problem under study. Thus, from a conservation point of view, it would be possible to provide a robust tool to allow managers and policy-makers to achieve their objectives.

The increase and improvement in the use of these models is mainly due to advances in the field of computing and the greater knowledge of ecological processes. The potential of modern computers to operate efficiently with a large volume of information and the availability of free software, have allowed new ways of approaching and studying the problems in many fields of science [10]. Among the great availability of modeling methodologies, Population Viability and Multi-agent models could be highlighted as the most frequently used [11]–[15].

Among models of computation, we highlight here the bio-inspired models. These models arise from the observation of processes in nature. The Population Dynamics P systems (PDP) models are a variant of P systems also known as multi-environment probabilistic P Systems with an active membrane [16] inspired by the structure and function of living cells [17]–[18]. These computational models have recently been applied to study population dynamics [16], [19], [20]. However, the great potential of PDP comes at a cost. PDPs are necessarily more complex in structure, so they are more difficult to analyze, understand and explain than traditional analytical models. Like other new generation models, a critical point is the problem of communication [21]. Analytical models are formulated mathematically and their description is usually complete and unambiguous. On the contrary, PDP models, as well as agent-based models, are more complex, but they use a language closer to the experts.

Emerging new generations of computational models can constitute useful tools, allowing the study of complex problems in a more affordable way. PDP models are at an early stage of expansion and thus it is necessary to establish a protocol for the design phase and application. The objective of this paper is to describe a protocol for the design and application of PDP models. We present two examples of applications of the protocol and summarize our experience, providing practical guidelines for its use. Finally, we discuss the pros and cons in the use of this tool in comparison with the multi-agent models currently used.

## Methods

### Population Dynamical P System

The PDP models (PDP systems) are a variant of P system models. The P system models are inspired by the functioning of cells. Cells are able to run multiple processes in parallel in a perfectly synchronized manner making them good candidates to be imitated for modeling complex problems.

A PDP system can be viewed as a cellular tissue in which each cell is within a special compartment called environment [16]. The cells have a particular structure hierarchy in which there is a skin membrane that defines and distinguishes the inside from the outside. In turn, inside a cell there are a number of hierarchically arranged membranes, where organelles or chemical substances capable of evolving according to specific reactions of the membrane may appear.

**Figure 1:**
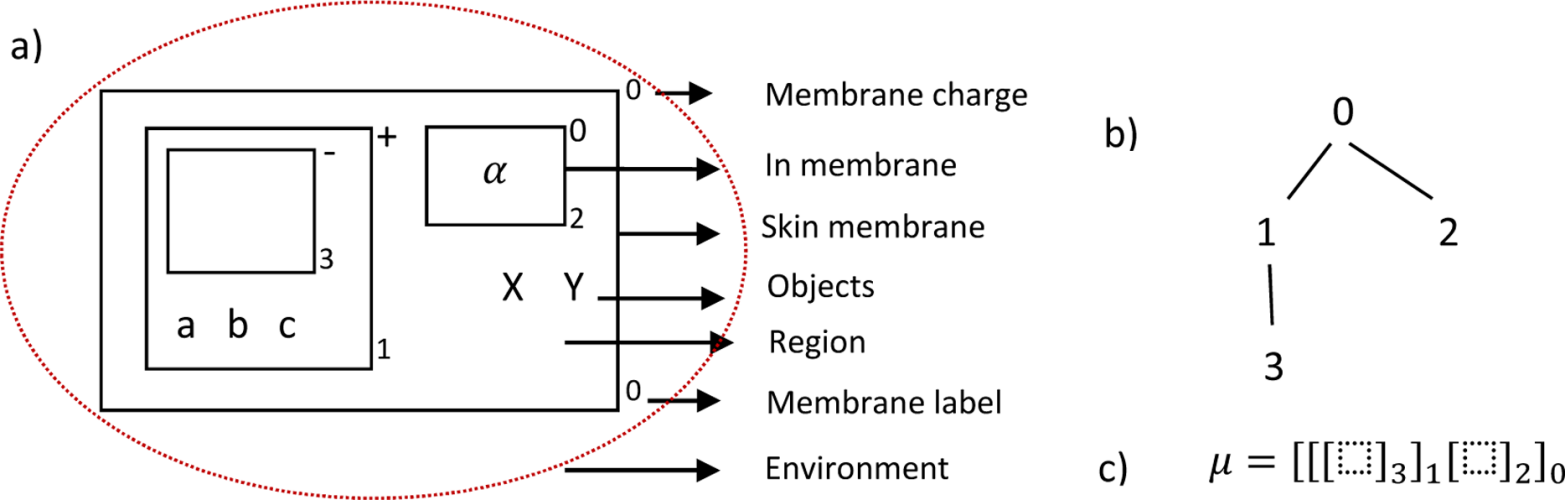
a) Representation and components of the cell, b) representation of the membrane structure using a rooted tree, and c) the analytical representation of membrane structure.

G. Păun [17] proposed an abstraction and graphical representation of the cell that allows the definition of P systems. A cell has associated a membrane structure consisting of several membranes arranged in a hierarchical structure inside a unique external membrane, the skin, (represented by an external rectangle) and delimiting regions (space in between a membrane and the immediately inner membranes). Regions contain objects and they can evolve according to given rules. (Figure 1).

All cells in the system have the same membrane structure, which can be formally described by a rooted tree (Figure 1), the external membrane is the father of inner membrane. Membranes are identified by labels that appear as subscripts on the membrane. To simplify the task of designing the model, it must be noted that membranes have electrical charges (positive, +, negative −, or neutral, 0).

A PDP model can be viewed as a collection of environments each of them containing a cell with the same membrane structure.

The basic components of the PDP system are:

- A set of environments that are connected among them according to some prefixed relation, and which can be formally described by a network.
- A membrane structure that provides the hierarchy among the different membranes that constitute the cell contained in each environment.
- A working alphabet that allows the representation of objects (individuals, resources, etc.) involved in the system under study. Individuals or objects that may be present in the environment are represented by using a specific alphabet contained in the work alphabet.
- A set of rules for cells that will enable the specification of the evolution of the objects inside and a set of rules for the environments that serve to specify how individuals can move from one environment to another, to generate values for variables that are correlated between environments and to generate objects whose multiplicity will depend on the environment.

The rules that govern the cells and the environments are particular mathematical expressions that are abstractions of the interactions that occur in the real system. Every rule consists of a left-hand side (where objects and conditions appear that must be taken in order to be executed and to facilitate the evolution of these objects), and a right-hand side in which there are objects that have been produced after the application of this rule. In the cells of the P systems, rules have the following syntax:
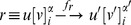



Where 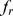 is a probabilistic function associated with the rule. If 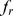 is the constant function equal to one, then we omit it.

If in an area delimited by membrane 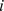, which possesses electrical charge 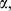 we find an object multiset 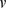 and in its father membrane we find an object multiset 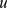, the rule can be applied with a probability 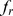. The application of this rule changes the polarization of the membrane from 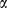 to 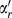 and the multiset of objects 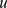 and 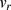 evolve to 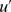 and 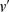, respectively.

Since there are different environments, there can be communication between them. When an object comes out of the skin membrane, and it is in the corresponding environment, then it can evolve according to environmental rules, which are of type:
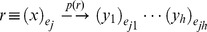



Object 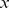 passes from environment 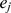 to environment 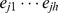 possibly modified into object 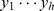, respectively. Function 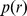 indicates the probability of executing the rule.

At some point, all the possible rules are applied in a maximal way, causing the P system to evolve and its configuration to change. A computation of the systems is a sequence of configurations, each of which is obtained from the previous configuration through a transition step.

We can establish a certain analogy between a PDP system and an ecosystem. On the one hand, an ecosystem corresponds to some physical space where there can be a number of distinct areas for certain characteristics (e.g., landscape, weather conditions). Within these areas, there are individuals whose development is conditioned by their own biological and demographic singularities. Individuals evolve simultaneously and interact and compete with each other and with the environment according to patterns or evolution rules. It is also possible that individuals can move from one area to another according to certain ecological restrictions (e.g., food, carrying capacity). Each of these areas can be identified as a different spatial environment of a PDP model and its contents can be specified as a cell having its own structure and its own life style traits. In that situation, we have to specify the rules that allow an individual to move from one environment to another, and the rules that apply within each of these areas (cells that make up the system).

In ecosystems, the processes are carried out simultaneously, synchronized and inter-related. The synchronization can be materialized by the biological cycles of the organisms that compose it. Therefore, we can assume that there is a global clock in the system.

## Results and Discussion

### Stages in Model Formulation: Establishing the Protocol

Once the analogy between the PDP and ecosystems has been described this bio-inspired computational paradigm can be used as a new framework for the study and analysis of ecosystem dynamics. The following will describe seven stages for obtaining a simulation tool based on PDP systems. The first four stages are common to any type of modeling. If the type of model used is a PDP the fourth stage has a specific design.

#### Stage 1: Defining and clearly limiting the objective proposed and the interest of the model

A series of questions must be answered. For example, what is the objective of the model? What will the outcome be? What information will be made available? What aspects can be addressed with the available means?

#### Stage 2: Description of the processes to be modelled as well as of the interaction between them and other processes

Once the purpose of the model has been clarified, the processes to take into account must be selected and the relationships between them must be established. In the first phase of modeling the most important elements are considered, and the rest will be introduced gradually until a model exists upon which the following phases can draw reliable conclusions from reality. PDP model systems are modular and therefore it is relatively simple to add new components.

#### Stage 3: The input of the model and the parameters involved are established

This stage is usually the most expensive one because it requires great effort. Complex models require a significant amount of information that is not centralized. Therefore, this step requires an exhaustive search for information since the final results will depend on the quality of data obtained.

#### Stage 4: Designing a model scheme that describes the sequencing and parallelization of the processes

At this stage we design a first draft of the algorithmic scheme that we want the model to capture, specifying the processes that are executed sequentially and those that will be executed in parallel. The outline structure responds to repeated execution cycles such that each of them represents a predetermined time interval.

Classic models are sequential, and thus the schemes are linear. Hence, in the process of carrying out the model, bifurcations may exist, however only one path can be chosen to achieve a final result. PDPs are individual models in the sense that each individual evolves independently, interacting and competing with other individuals. These models are synchronized at any given time and desynchronized at other times. Thus, at a given moment it is possible to execute different processes simultaneously, that is to say, it’s possible to execute at the same moment all paths derived by a bifurcation. In complex problems the scheme of PDP models will not be linear, so synchronization is essential.

#### Stage 5: Designing the model

**Figure 2:**
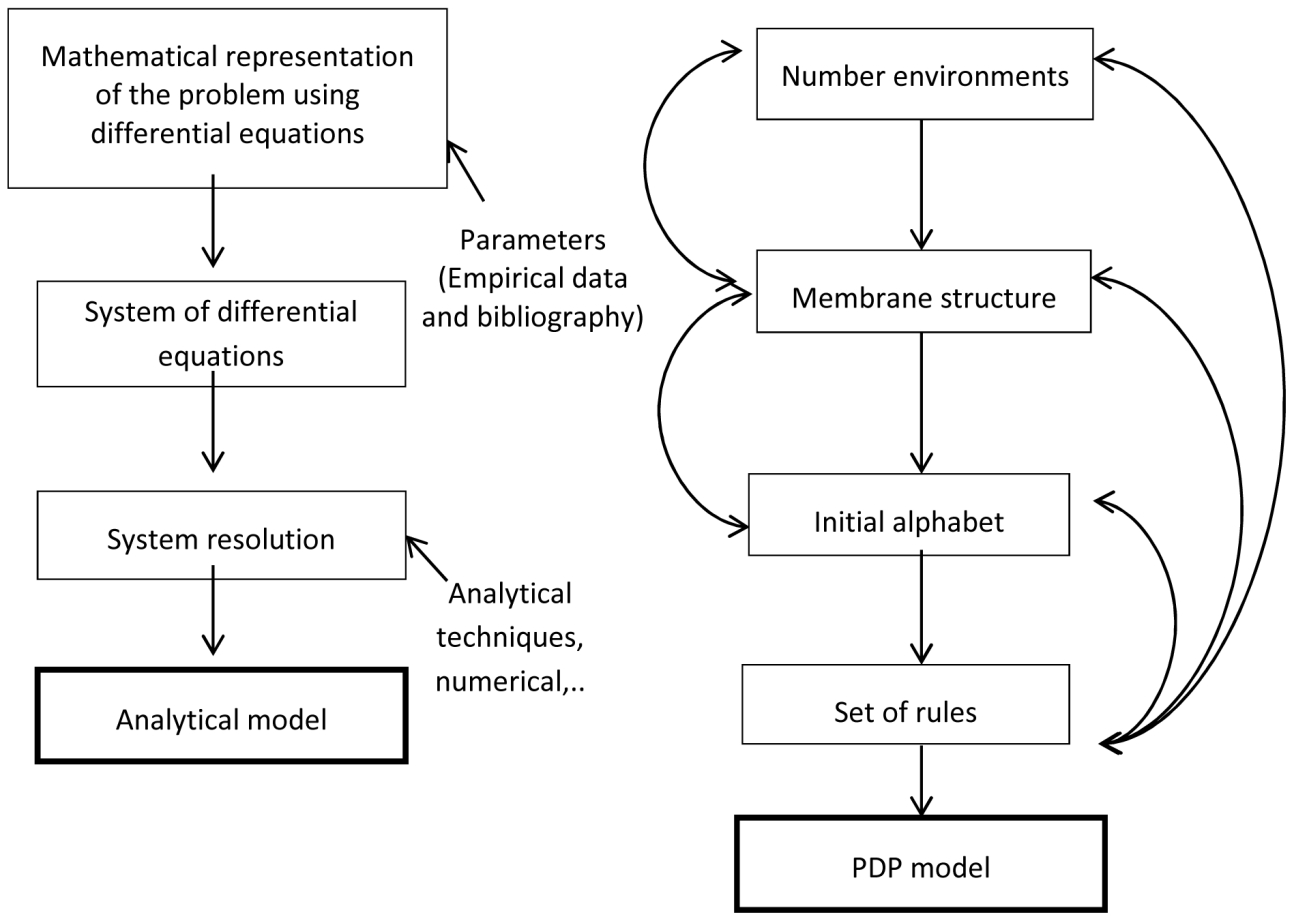
Conceptual representation of the main difference between classical model based on differential equations and PDP models.

At the beginning of this stage, all of the information needed to obtain the model is available. For classical models there is a standard and linear methodology and in some cases the application of sophisticated calculation techniques is required. In the case of the PDP model, the difficulty increases as the complexity and the power of the model increase. The steps to follow are fed back, such that it is often necessary to redefine them several times in order to obtain the final model (Figure 2). The PDP systems are very powerful probabilistic models, allowing simultaneous work with an unbounded number of spatial environments and species that operate in parallel and interact and compete with each other. They can take into account the simultaneous effect of environmental variables such as climatic risks of contagion effects of diseases, sudden changes in food availability, etc. The potential of these models involves a cost increase in the design phase of the model.

The criteria for choosing the components of the PDP models depend on the strategy adopted by the designer. However, below we suggest some guidelines especially useful to researchers who are new to this type of model.

1. The process begins by setting the number of environments that the model will contain. In the case that physical zones can be distinguished in the area of study, it is recommended that an environment for each zone is defined, only if we can guarantee the same biological parameter values in all environments. Otherwise, we recommend the use of a single environment.
2. The next step is to fix the structure of the membranes such that it is the same for all cells. It is advisable to begin modeling using two membranes, the skin membrane and an inner membrane. A simple structure allows the modeling of many problems. If this structure is not enough to capture the complexity of the problem, the number of membranes will be greater. One can increase the depth of the membrane structure by adding membranes within the inner membrane or by increasing skin membrane daughters. The second option usually carries a lower computational cost to facilitate the movement of objects.
3. Having defined the structure of the environments and the membranes, the next step is to associate an object with each of the actors involved in the problem, i.e., the input model. We can distinguish two types of inputs corresponding to the individuals forming the study population (animals, plants, etc.) and that correspond to the processes that determine the evolution of the population (weather conditions, food available, diseases, etc.). It is recommended that the objects associated with individuals are within the skin membrane at the initial moment. The items needed for the execution of the processes will be introduced into the environment if the values between environments are correlated, usual with climate variables.
4. It should bring order to the chaos which they apparently have the PDP models by synchronizing processes at certain times. In the simplest cases synchronization is obtained simply by evolution rules. In the case of complex models it is suggested that objects called counters that register the steps executed in the model are introduced, allowing the activation and deactivation processes to occur in a controlled manner.
5. The last step in defining the model is the formulation of the rules that will allow the evolution of objects. Following the scheme proposed in step 4, the rules are formulated by describing the processes that have been observed to take place in nature. The rules must be consistent, such that they are applied at the right moment, according to the structure of the cycles.
6. We can imagine the running of a PDP model as a box in which the ingredients that activate the execution and allow the evolution of the model are placed. This box is then closed and after a time the final product is obtained and analyzed. After the start of the model, no external intervention may occur, and therefore the correct definition of the rules is key to success of the model.
7. Although the rule set is unique and without priority between them, it is advisable to present the rules of the model grouped, so that each group corresponds to the rules to be applied in each step.
8. Researchers will use their own strategies and resources to implement the four previous actions. One way to achieve the final objective is to choose the model that minimizes the use of computational resources.

#### Stage 6: Graphical representation of the configurations that represent the execution of a cycle of the model

It is important to check that the rules are well-defined and consistent and that the model is synchronized, although the model has been described in the first five stages. Graphic representations allow the visualization of the steps that the model will execute and detect whether there has been an error in the design phase. These representations are particularly useful for understanding the model, because objects are displayed, as are their position and evolution.

#### Stage 7: Designing the simulator

The PDP systems are computational models, therefore a simulator must be designed in order for the model to be applied. Thus, computer simulators are necessary to facilitate the implementation of the model for different scenarios of interest. At present, free software, called MeCoSim, designed by the Natural Computing research group of Seville University, is available [22]. This is a visual environment that allows the configuration of inputs and outputs. The simulations are carried out using P-Lingua Core [23]. The input of MeCoSim are two files: *noma.xls* and *nomb.pli*. In the first, the menus and sub-menus of the simulator, data tables, the values of the parameters used by the model and the outputs in the form of tables and graphs of the simulator are defined. The second file, written in ASCI code, saves the model and defines the structure of membranes, the initial alphabet and rules.

The simulator must be able to reproduce the randomness of real processes where objects compete with other objects and are involved in several rules that run simultaneously. According to the algorithm used as an engine simulator, it may happen that there are variations in the results, especially when resources are scarce [21]. P-Lingua [23] is a special framework designed to simulate different models of P systems, particularly PDP systems. The algorithms used in the simulation have evolved to capture the randomness of the process and allow the distribution of resources to be carried out properly [16]. If there are objects used in multiple rules simultaneously, the distribution of objects is not trivial (see more details in [24]).

The great potential of the PDP model is that apparently complex problems, difficult to treat using classical models, can be modeled with ease. Such as problems that are modeled in the following case studies.

### The Case Studies: Application of PDP Models Following The Standard Protocol

This section will present two case studies, the first to be developed fully. The second, far more complex than the first, is shown to describe and discuss the way that the computational cost has been resolved such that it does not increase excessively.

#### Case 1 work presented in Russell et al. (2009)

The stages of the protocol described in the previous section will be applied to design a model based on an ecosystem presented in [25]. This work proposes a mathematical model based on systems of ordinary differential equations to study the dynamics of an ecosystem that consists of the interactions among birds, cats and rats. The objective is to control the bird population by the introduction and control of cats and rats. The designed model is applied to a hypothetical case of an island on which gadfly petrels (Pterodroma spp.) live. The model is described by a system of differential equations, which cannot be solved in a simple manner; for the resolution the authors use a non-standard scheme of finite differences.

Here, we present an alternative modeling methodology based on PDP models, which is much simpler and, moreover, does not require sophisticated techniques for resolution and implementation of the model.

The first three stages that are described below are exactly as the authors presented in [25].

#### Stage 1 Objective

The purpose is to present a model to estimate the dynamics of gadfly petrels on an island in the Pacific Ocean, under different scenarios controlled by humans. The scenarios are defined according to the introduction and population control of cats and rats.

There are basic biological parameters of gadfly petrels such as: sex ratio, mortality based on age, life expectancy, reproductive success, and number of offspring per reproduction. The parameters for the other species are taken from references or their values are fixed according to the experience and knowledge of experts.

#### Stage 2 Modeling processes

The processes to be modeled for the gadfly petrels are: Reproduction, natural mortality and predation. Food has not been considered as a limiting factor. Seven age-classes are considered: fledglings, five pre-adult age-classes and adults.

The processes to be modeled for the cats are: The population size (introduction and capture of animals) as controlled by humans and feeding. Cats can feed on mice, skinks and birds in their first years or as adults.

The processes to be modeled for the rats are: The population size (introduction and capture of animals) as controlled by humans, mortality due to hunting and feeding. Rats can feed on vegetation and birds at an early age.

**Figure 3:**
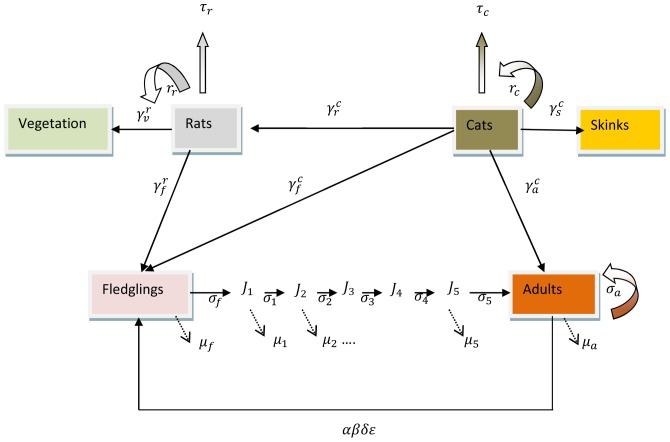
Conceptual representation of the age-structured differential predation model, 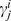 is the predation rate of population i on population j where 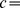
cats, 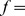
fledglings, 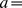 adult bird, 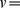
vegetation and 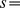
skinks. 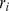 is the annual intrinsic growth rate of population *i*. For birds, 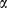 is the adult sex-ratio, 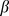 the proportion of breeding adults, 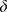 the adult pair fecundity, 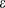 the number of clutches, 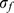 the fledgling survival, 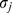 the juvenile survival and 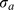 the adult survival. 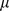 is the corresponding mortality where 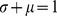 Russell *et al.* (2009).

Figure 3 reproduces the scheme of the problem to be modeled presented by [25].

#### Stage 3 Input of model and parameters to be taken into account

The input of the model is the initial population size and the parameters of the model obtained by [25] (Table 1).

**Table 1:** Biological parameters used for the model (Russell *et al.* 2009).

Parameter	Symbol	Value
**Annual demographic parameters**		
Adult sex-ratio	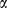	0.5
Proportion of adults breeding	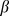	0.9
Adult pair fecundity	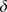	1
Number of clutches	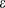	1
Sub-adult classes	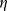	5
Fledgling mortality	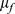	0.34
Sub-adult mortality	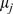	0.2
Adult mortality	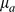	0.07
Expected adult lifetime (years)	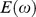	18
Maximum adult lifetime (years)	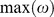	48
Bird growth rate	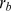	0.03
Bird annual reproduction	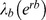	1.04
Adult bird carrying capacity	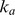	100.000
Cat growth rate	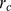	0.25
Rat growth rate	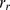	4.00
*Annual per capita predation rates*		
Cats on rats	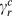	244
Cats on adult birds	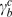	70
Cats on fledglings	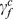	22
Cats on alternative (skinks)	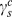	150
Rats on fledglings	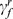	8
Rats on alternative (vegetation)	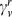	300
*Alternative food sources*		
Skinks (cat alternative food)	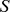	100.000
Vegetation (rat alternative food)	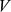	100.000

#### Stage 4 Sequencing and parallelization of the processes

**Figure 4:**
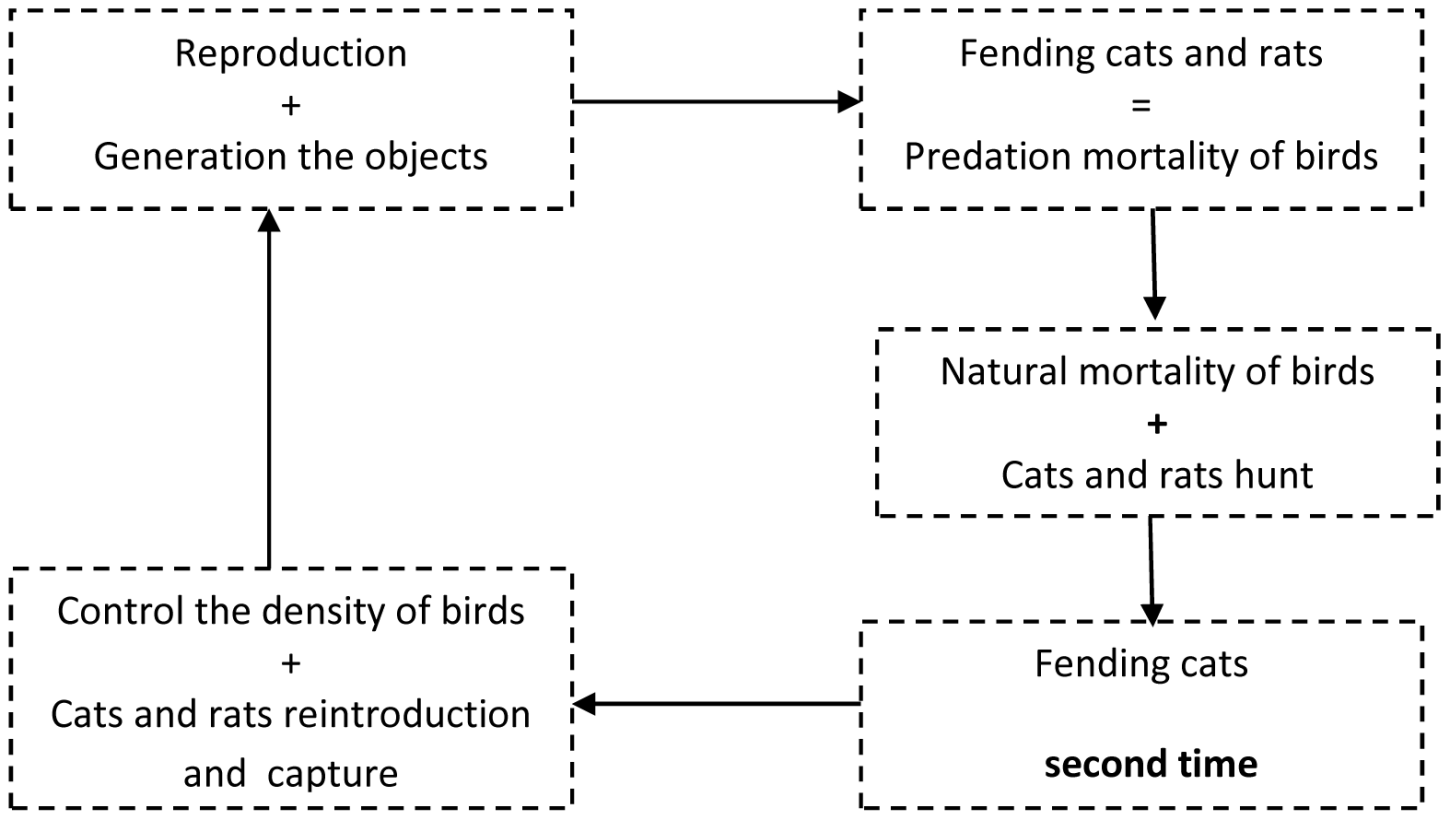
Scheme proposed for the PDP model. The loop is formed by five modules that are applied sequentially. In three of the modules, two processes are applied in parallel. The objects associated with each of the animals evolve simultaneously in the different modules.

The sequencing proposed for the modeling algorithm is shown in Figure 4. It consists of a sequence of five modules, in some of which more than one process is executed in parallel and, in all cases, individuals evolve individually but simultaneously (i.e., each individual has its own evolution and all individuals evolve at the same time).

### Stage 5 Designing of the Model

1. The ecosystem studied is an island that has no differentiated areas so we define it as a single environment.
2. The model will be tested with the simplest structure: 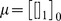 the skin and one in membrane.
3. The variable inputs of the model are: birds, cats and rats. The skinks and vegetation are fixed contributions of ecosystem. The density of birds should be controlled such that it does not exceed the maximum load of the island. According to this information, the initial alphabet is:

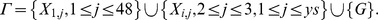



Each animal is associated with an object 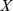 with a pair of indices. The first represents the species (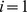
bird, 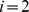
cat and 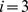
rat) and the second indicates the animal’s age for birds. In the case of cats and mice the second index will be used to record the year of simulation. This will facilitate human intervention in these species. Object 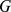 will allow us to create objects that will be used to control the density of birds, and the objects that are associated with vegetation and skinks.

1. Following the scheme proposed in stage 4 we will express mathematically the evolution rules for the processes involved in each module. It should be noted that the output of one module is the input of the next module. The evolution rules are as follow and the parameters used appear in Table1.

**First configuration: Reproduction + object generation,** The object 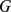 allows the generation of objects 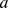 to be used to control the maximum load capacity of gadfly petrels,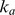, Objects 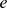 are used to generate randomness in the load of the animals. According to Russell et al. (2010) the number of skinks and the vegetation available each year is taken as a constant value equal to 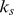 and 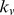, respectively.
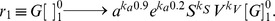



The following rules belong to the process of reproduction of gadfly petrels. The objects 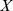 associated with animals that do not reproduce evolve to 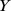 objects, while objects associated with reproducing animals, change to objects 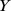 and create new objects 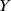 with the second subscript, age measured in years, equal to 0.
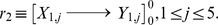


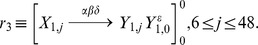


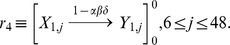



In the case of cats and rats the exact reproduction process does not apply because existing information is the rate of population growth. This is done in a different way: if growth is below 1, then cats, if greater than 1, then rats.
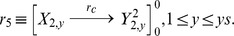


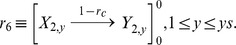


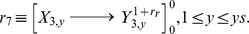




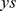
 means years simulated. That is an input of the model.

#### Second configuration: Fending cats and rats or predation, mortality of birds

In this configuration step a random number of objects 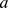 is generated and therefore the maximum load of gadfly petrels.
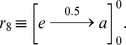


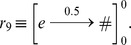



Cats can be fed fledglings, adult birds, rats and skinks, the amounts needed are respectively, 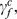 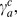 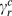 and 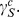


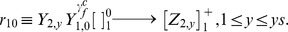


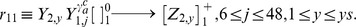


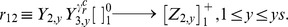


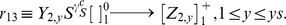



The rats may feed on fledglings and vegetation. The amounts needed are respectively 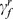 and 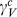.
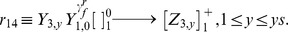


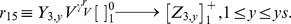



Objects differ for cats and rats that have eaten from those who have not been able to eat. The first evolves to objects of type 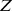 and enters the membrane 1 that changes its polarization. In the case that cats and rats do not exist or that there was no food for these two species, membrane 1 does not change its polarization, creating an inconsistency in the model. To avoid this, the model always applies the following rule.
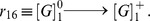



#### Third configuration: Natural mortality of birds and cats and rats capture

Rules belong to the process of natural mortality of gadfly petrels. Some of the objects of type 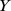 associated with animals that die disappear while the objects associated with surviving animals evolve into 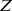 objects that enter into the membrane 1.
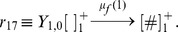


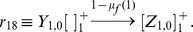


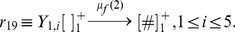


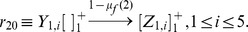





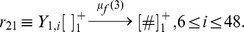


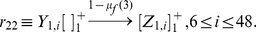



This step is used for objects 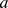 that will permit the control of the maximum load of the gadfly petrels to enter into the membrane 1. The objects 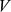 and 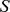 are dissolved because the feeding process in which they are involved has already been run.
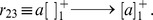


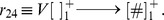


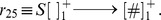



Rats have not eaten; have no opportunity to find food, while cats may still feed on rats. Feeding processes of rats and cats will be in parallel. Therefore the objects 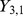 disappear while objects 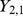 come into the membrane 1.
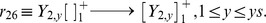


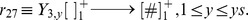



To ensure the consistency of the model the following rule is applied:
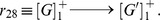



#### Fourth configuration: Cats that eat rats, which have eaten previously

If there are rats, cats can be fed and therefore objects 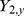 evolve.
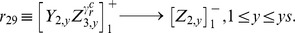


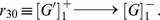



#### Fifth configuration (Initial configuration) Control density birds retire cats and rats and restore initial configuration

This controls the density of adult birds and dissolves the objects associated with animals that have reached the maximum age.
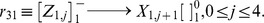


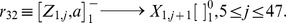


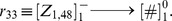



It takes some cats and rats.
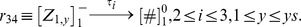




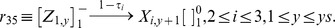
 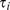 animals withdrawn.

Cats that have not been able to eat, die.
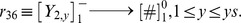



Object 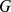 is restored to restart the loop.
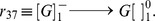



Rule that is applied in the new loop.
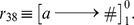



The proposed model consists of 38 types of rules involving 248+58⋅*ys* rules (*ys* number of years to simulate).

#### Stage 6 Graphic representation of the model configurations

**Figure 5:**
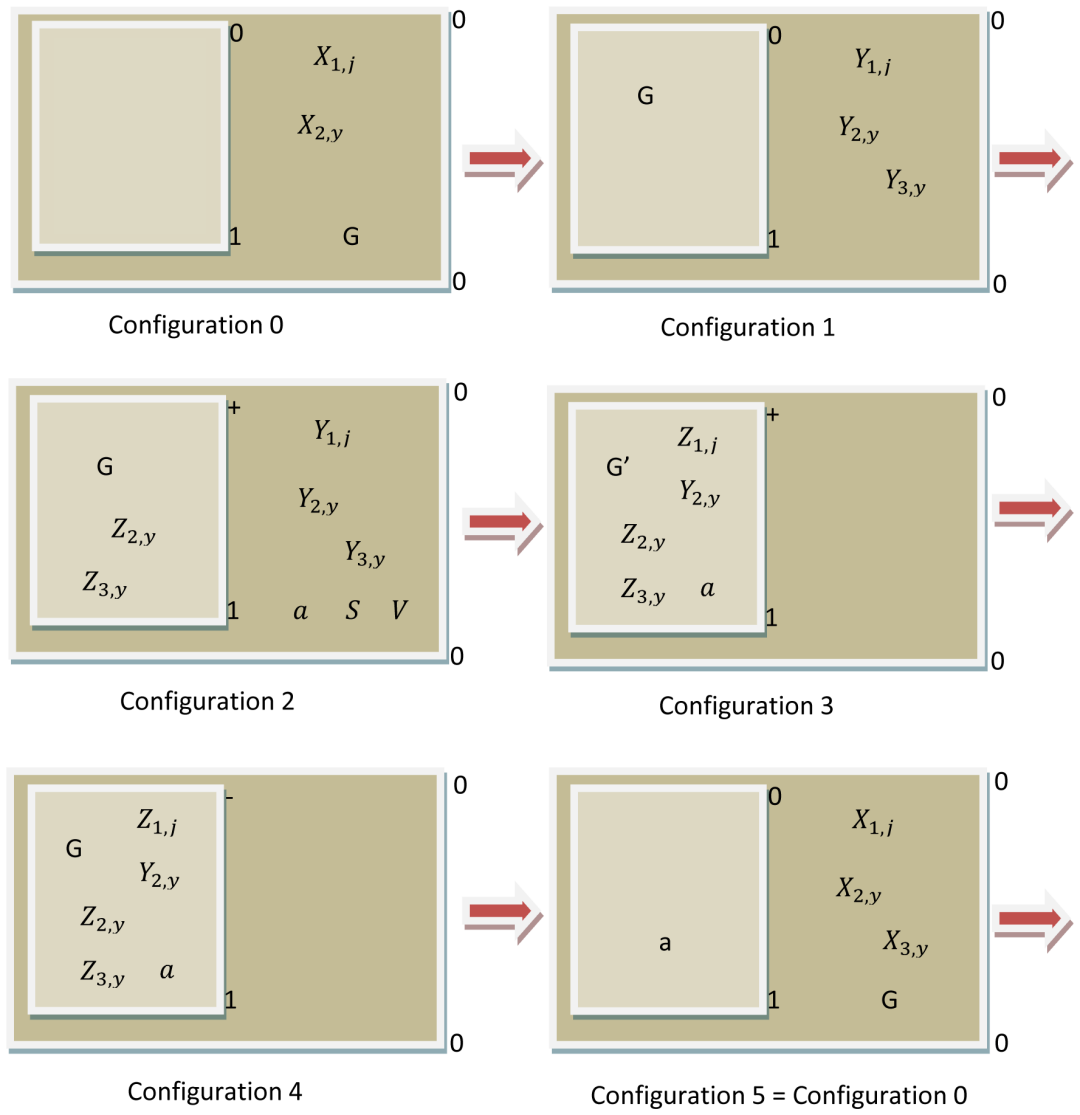
Types of configurations that appear in the execution of a loop of the model. The representation shows the types of objects that appear.

Starting from the initial configuration and applying the rules that were presented at stage 5, the different configurations for the execution of a loop corresponding to the passage of one year are obtained sequentially (Figure 5). Each module has a configuration and usually a module contains more than one configuration.

#### Stage 7 Defining a simulator to run the model

**Figure 6:**
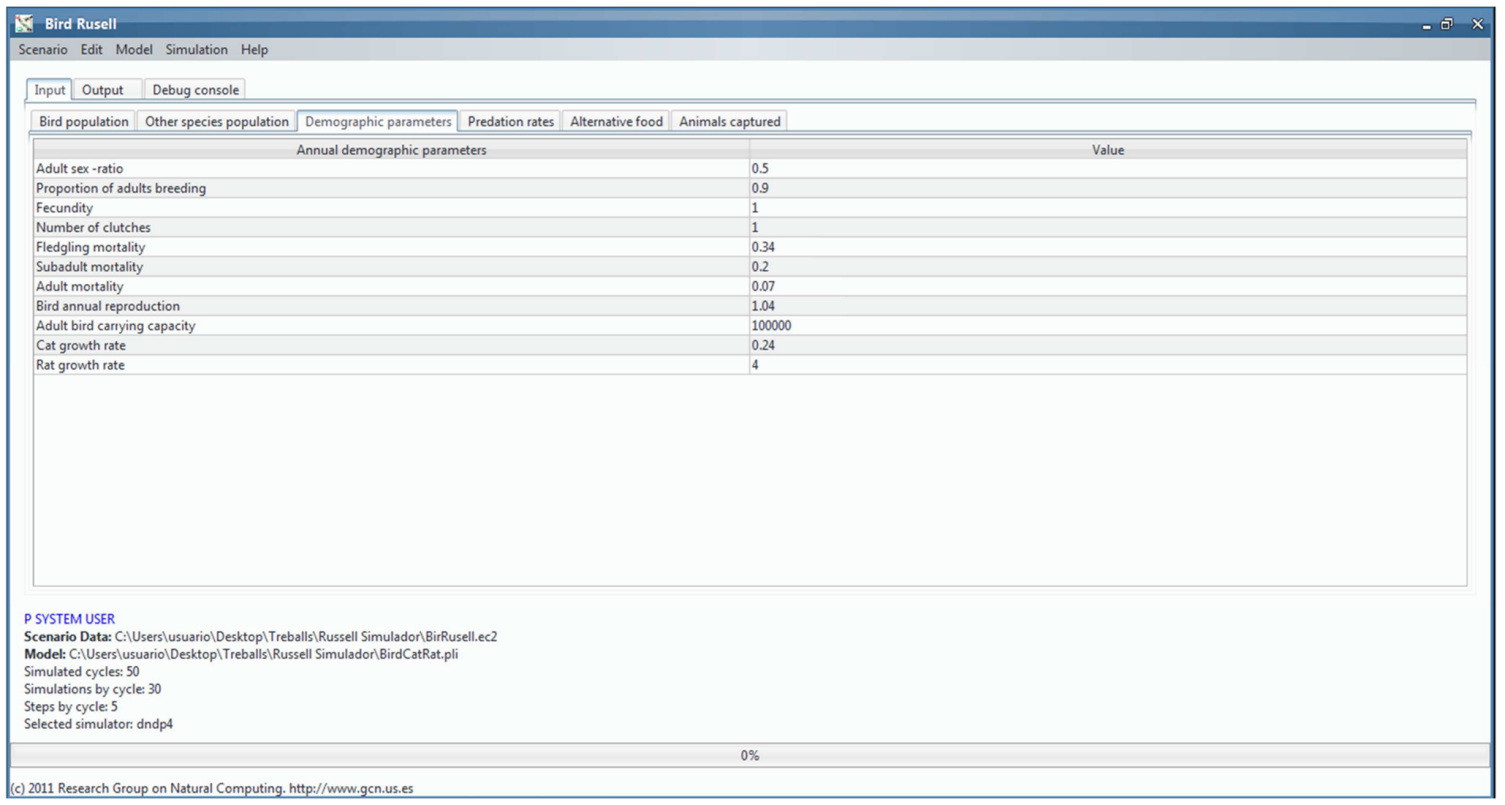
Screen of the simulator obtained using MeCoSim showing the demographic parameters. The user can change the values directly in the simulator placed in the box, which instantly tells us the evolution of the ecosystem by varying the starting scenario.

**Figure 7:**
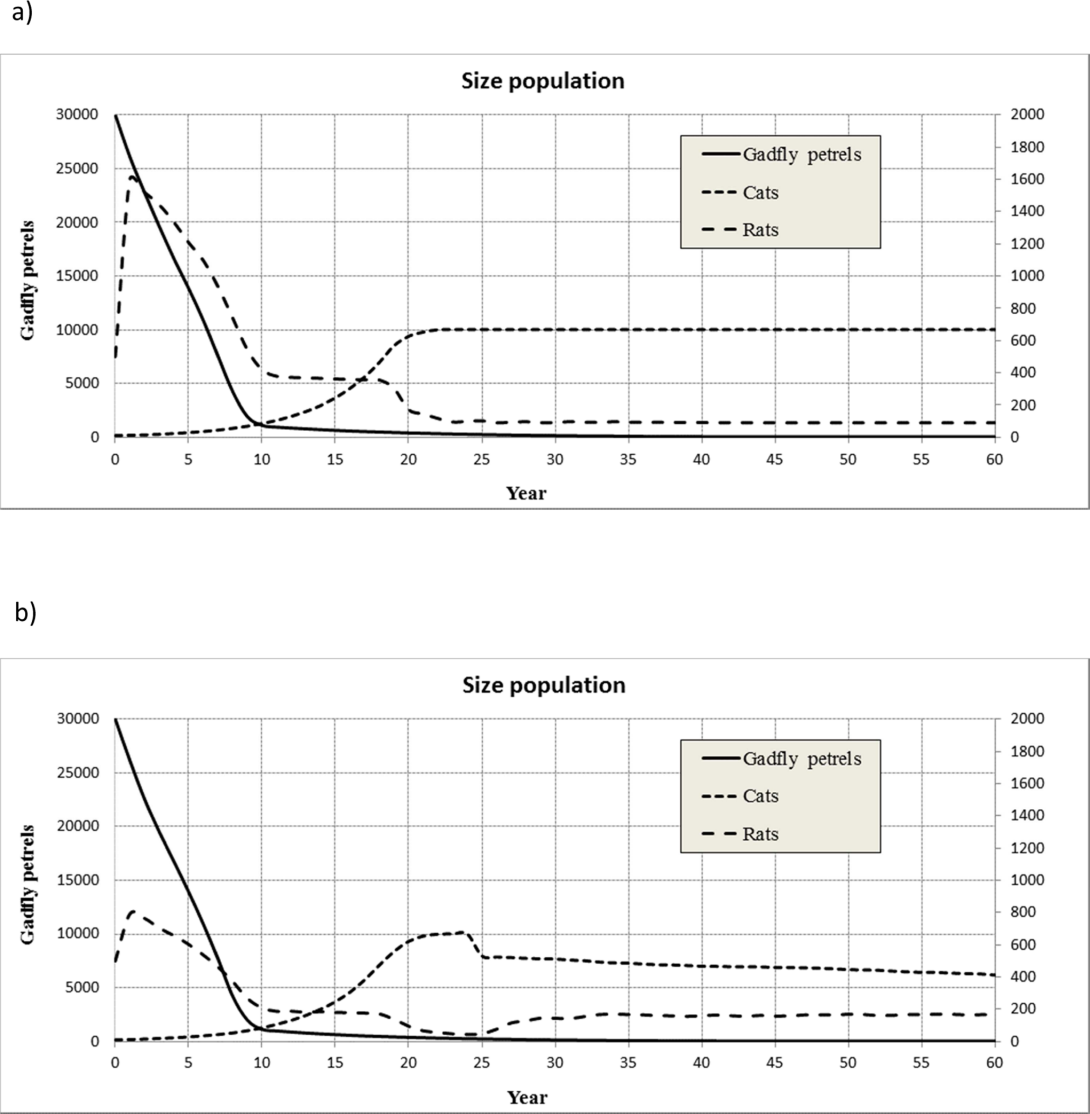
Population trend of gadfly petrels, cats and rats. The simulated scenario has been: gadfly petrels: 30 000, cats: 10 and rats: 500. The biological parameters used are shown in Table 1. a) Without human intervention, b) 50% of rats captured annually and from year 25 the 20% of cats are removed annually.

Once the model is defined, the next step is to define a simulator that allows the efficient running of the model. A software tool that allows the management of the model to predict the dynamics of gadfly petrels will be developed. MeCoSim (http://www.p-lingua.org/mecosim) was used to design the simulator interface. Figure 6 shows a screen input and Figure 7 shows a graphic representation of the results.

Input values (i.e., parameters and value variables of the model) are introduced directly into an interface of the simulator. When we want to study the behaviour of the model in a concrete scenario, we simply need to change the input values in this interface.

### Case 2 Work Presented in Margalida & Colomer (2012)

In [26], the PDP model is used to study the population dynamics of four avian scavengers (European griffon vulture *Gyps fulvus*, Egyptian vulture *Neoprhon percnopterus*, bearded vulture *Gypaetus barbatus* and cinereous vulture *Aegypius monachus*) in northern Spain under different food available scenarios.

#### Stage 1 Objective

In Europe, avian scavenger conservation depends on changes in the health regulations that affect the availability of food provided by the carcasses of domestic animals [27]–[29]. Given this backdrop, the goals were to design a model that simulates the population dynamics of the four species under different trophic availability scenarios.

#### Stage 2 Modeling processes

The problem is complex given the breadth of the study area, subdivided into 10 zones with different avian scavenger densities and with a large number of actors (domestic ungulates, wild ungulates, supplementary feeding sites and avian scavengers) and processes involved. In a simplified manner:

- Four species of scavengers coexist that feed on biomass provided by domestic and wild ungulates who share territory. Interspecific hierarchies exist in access to food by scavenging.
- There are six species of wild ungulates and three domestic ungulates; a portion of the domestic ungulate population is nomadic, so this population undergoes seasonal variability (greater availability of food resources in the summer). Some of the biological parameters of the species depend on the time of year, so the model should include seasonality.
- There are 20 artificial feeding stations distributed among the 10 areas with biomass input variable depending on the time of year.
- In addition to domestic and wild ungulates forming the basis of the diet of the avian scavengers, in the study area other small animals also provide complementary food (mainly for Egyptian and bearded vultures).
- Each zone supports a maximum load per species.
- There are some species of wild ungulates of hunting interest that in some cases is biased toward a particular sex (males as in the case of trophy hunting). Generally most of the carcass remains in the field are available to avian scavengers.
- In the case of a lack of resources, space or food, the scavengers can move to other areas or can even look for food in areas peripheral to the study area. Individuals can move beyond an area in search of food or space.

The processes to be modeled are: reproduction, natural mortality, hunting mortality, feeding (energetic requirements), carrying capacity and foraging movement among areas.

#### Stage 3 Input of model and parameters to be taken into account

The input of the model are the populations of different species in each of the areas, the biological parameters of each species, the external input of biomass by humans at the supplementary feeding sites and the network of the possible foraging movements among zones [26].

The output of the model includes predictions of population size for each species and year simulation and biomass available in the form of bones and meat that all ungulates provided over each area and year.

#### Stage 4 Sequencing and parallelization of the processes

**Figure 8:**
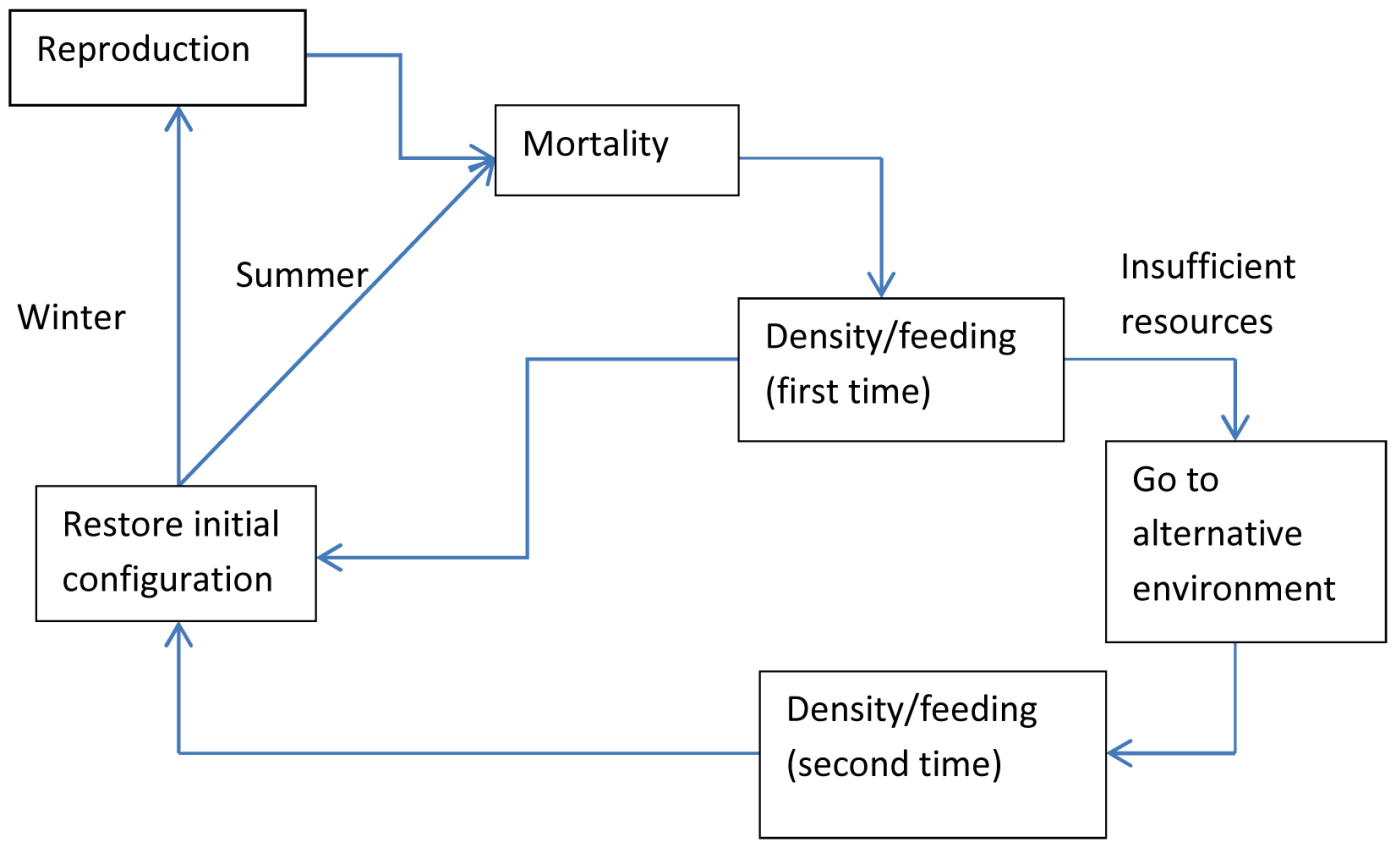
Scheme proposed for the model by Margalida & Colomer (2012).

The scheme proposed for the model [26] is reproduced in Figure 8.

#### Stage 5 Designing of the model

1. The ecosystem consists of 10 zones and the parameters are constant in all of them, such that initially the problem can be designed with 10 environments. In the model proposed in [26], there are 11 defined environments, 10 corresponding to the 10 natural areas and the eleventh is defined as a virtual environment to simplify and reduce the computational cost of modeling movements when resources are lacking.
2. The structure of membranes in the case of non-seasonality may be 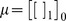, given that in our case we differentiate two periods (winter and summer), we need to double the structure. The final structure must be contained in a single skin membrane and is 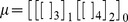. In the membrane labeled with the value 1 processes for the summer are carried out, while those labeled 2 correspond to the winter. The membranes labeled 3 and 4 are used to carry out specific process, in this case the mortality process.
3. The input variables of the model are existing individuals of each species in each area. External contributions made in the feeding stations and the complementary biomass provided by small animals are fixed in the ecosystem. The density of each species should be controlled such that they do not exceed the maximum load of the subarea. According to this information the initial alphabet work is:

Membrane labeled with 0:
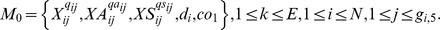



Membrane labeled with 1 and 2: 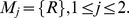


Membrane labeled with 3 and 4: 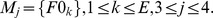


Each wild animal is associated with an object 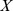 with a pair of indices. The first represents the species and the second indicates the individual’s age. In the case of domestic animals the object is 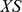 for the animals associated with transhumance and 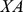 for the rest. 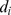 will control the maximum density, and 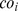 the time of year (summer 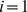, winter 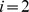). 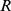 is a counter that is used for synchronization and finally the object 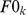 allows objects associated with the fixed biomass provided by the ecosystem to be generated.

1. Following the scheme proposed in stage 4, 209 rule types were defined [26].

#### Stage 6 Graphic representation of the model configurations

The execution of a loop (Figure 8) involves 20 configuration steps. The passage of a year involves running the loop twice and thus there are 40 configurations in a year. In [26], the 20 steps involving the execution of the loop are detailed and graphically discussed. In this case the model consists of six modules, and therefore there is no biunivocal relationship between modules and configurations as in the case presented above.

## Conclusion

PDP models can relatively easily treat complex problems considered untreatable using models based on differential equations and can simplify the modeling for treatable problems with differential equations as shown in case 1. Models are modular allowing us to begin by solving a very basic problem and increasing its complexity step by step. Thus, the process introduced to build the model can be retrospectively improved by comparing the results obtained with the actual trend observed (i.e., population dynamics trend in the case of modeling an ecosystem [19]). Hence, this allows researchers to directly modify the different values of the parameters on the screen and to quickly see if the results that the simulator provides are correct, which was not possible with traditional methods based on differential equations.

The great advantage of computational modeling is i) their ability to manage large volumes of related information, ii) the flexibility of these models to enable the increase in variables (i.e., number of species) without the need for modifying the model [27], iii) the capability to simultaneously model a large number of species that share the same space and their interaction with the environment, iv) the possibility of implementing spatial components in the ecosystem. PDP and multi-agent models have a lot of commonalities such as they both allow the study of complex problems with different interacting agents (processes). In the case of multi-agents, it is necessary to sequence the process whereas this is not necessary in PDP, and the interacting processes can run in parallel. This constitutes an important advantage to PDP models compared to multi-agent models, as not all real-world problems can be sequenced. For example, in the case of population dynamics of aquatic ecosystems in which the initiation of breeding process are modulated by thermic conditions (i.e., as in zebra mussels, *Dreissena polymorpha,* in which breeding extends over several months), eggs, larvae and pre-adults can occur simultaneously developing different processes at the same time. It is possible to solve this case using PDP models but not through multi-agent models.

The hierarchical structure of PDP models simplifies the prioritization and synchronization of processes and therefore facilitates the modeling and ease of implementing spatial components in the ecosystem. PDP models can be considered as a set of multi-agent models that are capable of communicating and interacting. Therefore they are more potent from a computational point of view. A simple problem, such as the one presented, is considered as complex by the authors [25], and can be modelled easily and quickly by researchers who begin with this new type of modeling. In contrast, to model complex systems [16], [20], [26], [30], [31] in which the ecosystem is formed by various environments and species interacting and competing for resources, an experienced researcher familiar with these models is necessary.

Today we have very powerful computers, capable of storing and managing large amounts of information. If we also consider the many free software packages that exist and the use of computer programming professionals capable of developing software according to specific needs, computer models can be very appropriate methods for studying complex problems.

All of these advances and resources that are at our disposal can be used to answer many outstanding questions. Adapting a new technique, however, involves a change in mindset. While traditionally we associate the word “model” with analytical expressions, we must begin to think of computational modeling not based on these expressions, but based on algorithms and the management of information and knowledge [32]. As a result, the applicability of executable models in ecological processes studies constitutes a potential and useful tool allowing us to represent complicated chains of events that until now were untreatable.

## References

[1] Sinclair ARE, Mduma S, Brashares JS (2003) Patterns of predation in a diverse predator–prey system. Nature 425: 288–290. 13679915 10.1038/nature01934

[2] Bascompte J, Melia C, Sala E (2005) Interaction strength combinations and the overfishing of a marine food web. Proc Natl Acad Sci USA 102: 5443–5447. 15802468 10.1073/pnas.0501562102 PMC556268

[3] Ives AR, Cardinale BJ, Snyder WE (2005) A synthesis of subdisciplines: predator–prey interactions, and biodiversity and ecosystem functioning. Ecol Lett 8: 102–116.

[4] Paine RT (1980) Food webs: linkage, interaction strength and community infrastructure. J Anim Ecol 49: 667–685.

[5] Bascompte J (2009) Disentangling the web of life. Science 325: 416–419. 19628856 10.1126/science.1170749

[6] Kendall BE, Bjornstad O, Bascompte J, Keitt THD, Fagan WF (2000) Dispersal, environmental correlation, and spatial synchrony in population dynamics. Am Nat 155: 628–636. 10777435 10.1086/303350

[7] Ostfeld RS, Keesing F (2000) Pulsed resources and community dynamics of consumers in terrestrial ecosystems. Trends Ecol Evol 6: 232–237. 10.1016/s0169-5347(00)01862-0 10802548

[8] Ings TC, Montoya JM, Bascompte J, Blütgen N, Brown L, et al (2009) Review: Ecological networks-beyond food webs. J Anim Ecol 78: 253–269. 19120606 10.1111/j.1365-2656.2008.01460.x

[9] Bastolla U, Fortuna MA, Pascual-García A, Ferrera A, Luque B, et al (2009) The architecture of mutualistic networks minimizes competition and increases biodiversity. Nature 458: 1018–1020. 19396144 10.1038/nature07950

[10] Reshef DN, Reshef YA, Finucane HK, Grossman SR, McVean G, et al (2011) Detecting novel associations in large data sets. Science 334: 1518–1524. 22174245 10.1126/science.1205438 PMC3325791

[11] Brook B, Cannon R, Lacy R, Mirande C, Frankham R (1999) Comparison of the population analysis packages GAPPS, INMAT, RAMAS and VORTEX for the whooping crane (Grus americana). Anim Conserv 2: 23–31.

[12] Mullon C, Cury P, Shannon L (2004) Viability model of trophic interactions in marine ecosystems. Nat Res Model 17: 71–102.

[13] Bousquet FC, Le Page C (2004) Multi-agent simulations and ecosystem management: a review. Ecol Model 176: 313–332.

[14] Grimm V, Revilla E, Berger U, Jetsch F, Mooij W, et al (2005) Pattern-oriented modeling of agent-based complex systems: lessons from ecology. Science 310: 987–991. 16284171 10.1126/science.1116681

[15] McGowan CP, Runge MC, Larson MA (2011) Incorporating parametric uncertainty into population viability analysis models. Biol Conserv 144: 1400–1408.

[16] Colomer MA, Margalida A, Sanuy D, Pérez-Jiménez MJ (2011) A bio-inspired computing model as a new tool for modeling ecosystems: the avian scavengers as a case study. Ecol Model 222: 33–47.

[17] Păun G (1998) Computing with membranes. J Comput Syst Sci 61: 108–143.

[18] Păun G, Rozenberg G, Salomaa A (2010) *The Oxford Handbook of Membrane Computing.* Oxford: Oxford University Press.

[19] Cardona M, Colomer MA, Pérez Hurtado I, Pérez Jiménez MJ, Sanuy D, et al (2009) Modelling ecosystems using P Systems: the Bearded Vulture, a case study. LNCS 5391: 137–156.

[20] Margalida A, Colomer MA, Sanuy D (2011) Can wild ungulate carcasses provide enough biomass to maintain avian scavenger populations? An empirical assessment using a bio-inspired computational model. PLoS One 6: e20248. 21629647 10.1371/journal.pone.0020248 PMC3101228

[21] Grimm V, Berger U, Bastiansen F, Eliassen S, Ginot V, et al (2006) A standard protocol for describing individual-based and agent-based models. Ecol Model 198: 115–126.

[22] Pérez-Hurtado I, Valencia L, Pérez-Jiménez MJ, Colomer MA, Riscos A (2010) MeCoSim: A general purpose software tool for simulating biological phenomena by means of P Systems. In: K Li, Z Tang, R Li, AK Nagar, R Thamburaj (eds.) Proceedings 2010 IEEE Fifth International Conference on Bio-inspired Computing: Theories and Applications: 637–643. Vol 1. Changsha: IEEE Press.

[23] Díaz D, Pérez-Hurtado I, Pérez-Jiménez MJ, Riscos A (2009) A P-lingua programming environment for Membrane Computing. LNCS 5391: 187–203.

[24] Martínez-del-Amor M A., Pérez-Hurtado I, García-Quismondo M, Macías-Ramos L F., Valencia-Cabrera L et al.. (2012) DCBA: Simulating population dynamics P systems with proportional object distribution. 13th International Conference on Membrane Computing (CMC13), Budapest, Hungary, p.291–310.

[25] Russell JC, Lecomte V, Dumont Y, Le Correa M (2009) Intraguild predation and mesopredator release effect on long-lived prey. Ecol Model 220: 1098–1104.

[26] Margalida A, Colomer MA (2012) Modelling the effects of sanitary policies on European vulture conservation. Sci Rep 2: 753. 23082243 10.1038/srep00753 PMC3475340

[27] Donázar JA, Margalida A, Carrete M, Sánchez-Zapata JA (2009) Too sanitary for vultures. Science 326: 664. 10.1126/science.326_664a 19900914

[28] Margalida A, Donázar JA, Carrete M, Sánchez-Zapata JA (2010) Sanitary versus environmental policies: fitting together two pieces of the puzzle of European vulture conservation. J Appl Ecol 47: 931–935.

[29] Margalida A, Carrete M, Sánchez-Zapata JA, Donázar JA (2012) Good news for European vultures. Science 335: 284. 10.1126/science.335.6066.284-a 22267790

[30] Colomer MA, Pérez-Hurtado I, Pérez-Jiménez MJ, Riscos A (2011) Comparing simulation algorithms for multienvironment probabilistic P system over a standard virtual ecosystem. Nat Comp 11: 369–379.

[31] Cardona M, Colomer MA, Margalida A, Pérez-Hurtado I, Pérez-Jiménez MJ, et al (2010) A P-System based model of an ecosystem of some scavenger birds. LNCS 5957: 182–195.

[32] Fisher J, Henzinger TA (2007) Executable cell biology. Nature Biotech 25: 1279–1249. 10.1038/nbt1356 17989686

